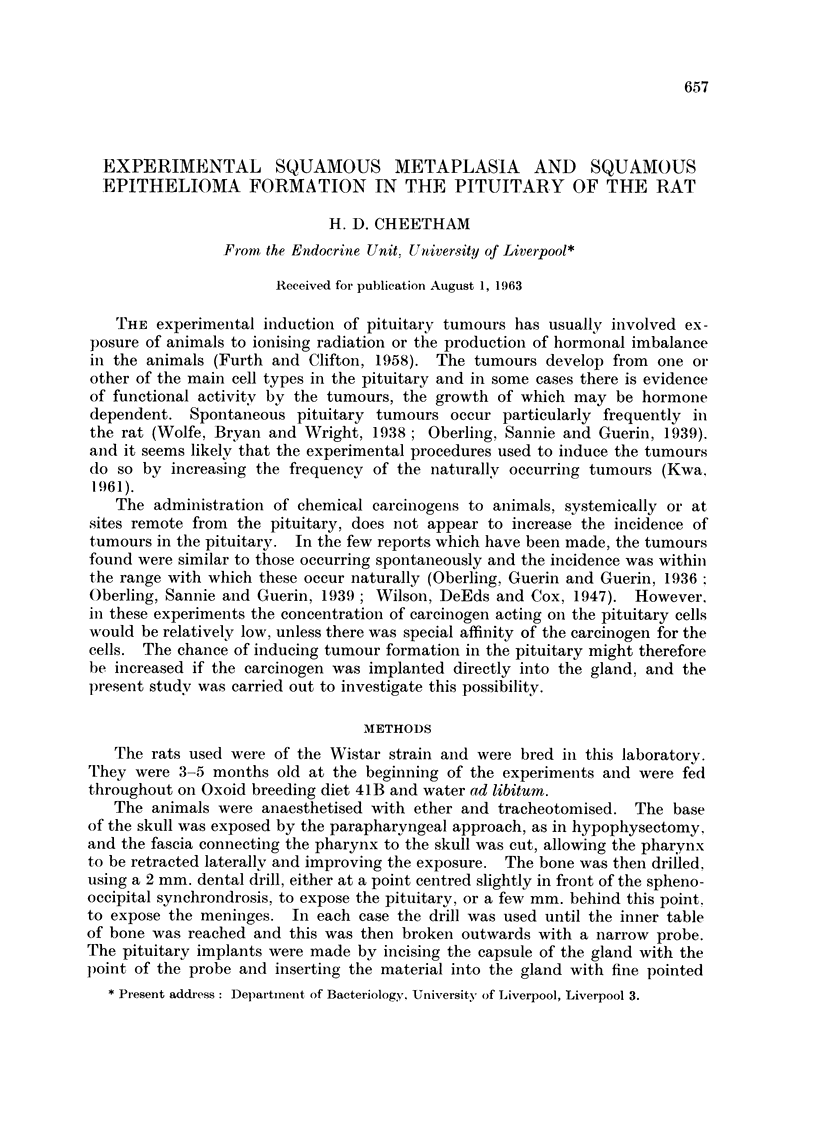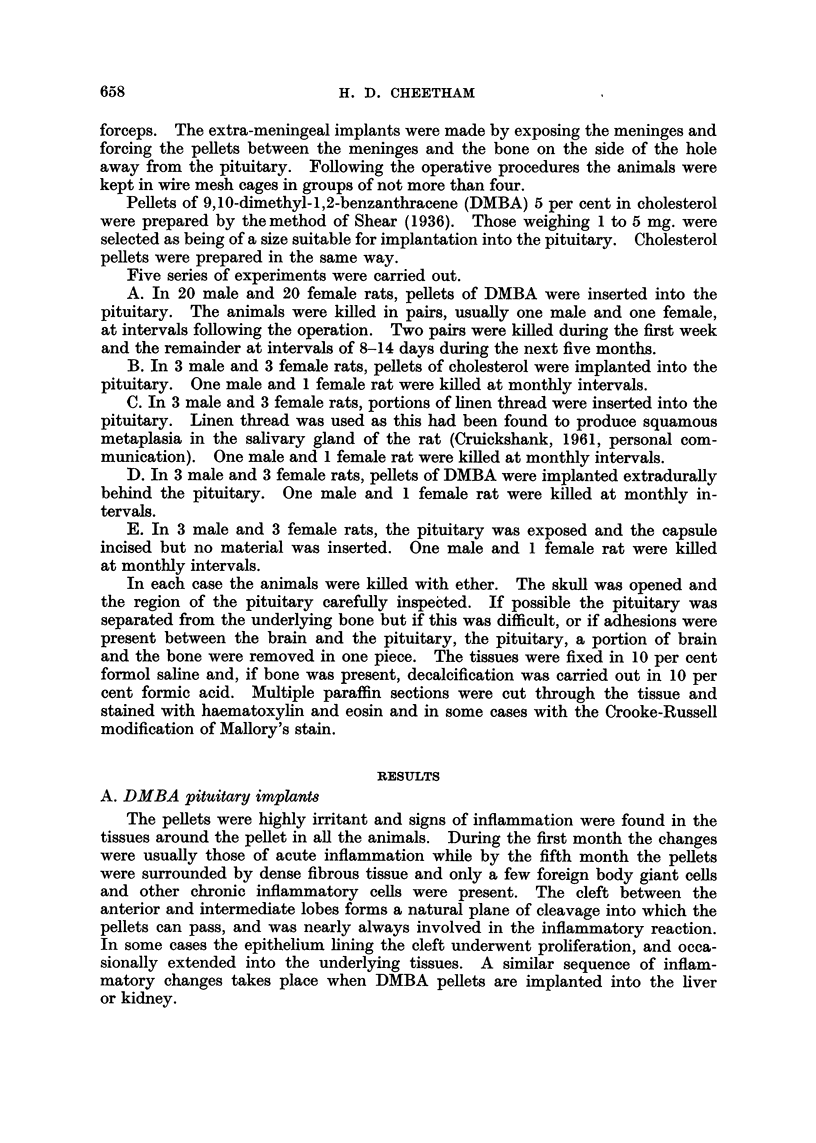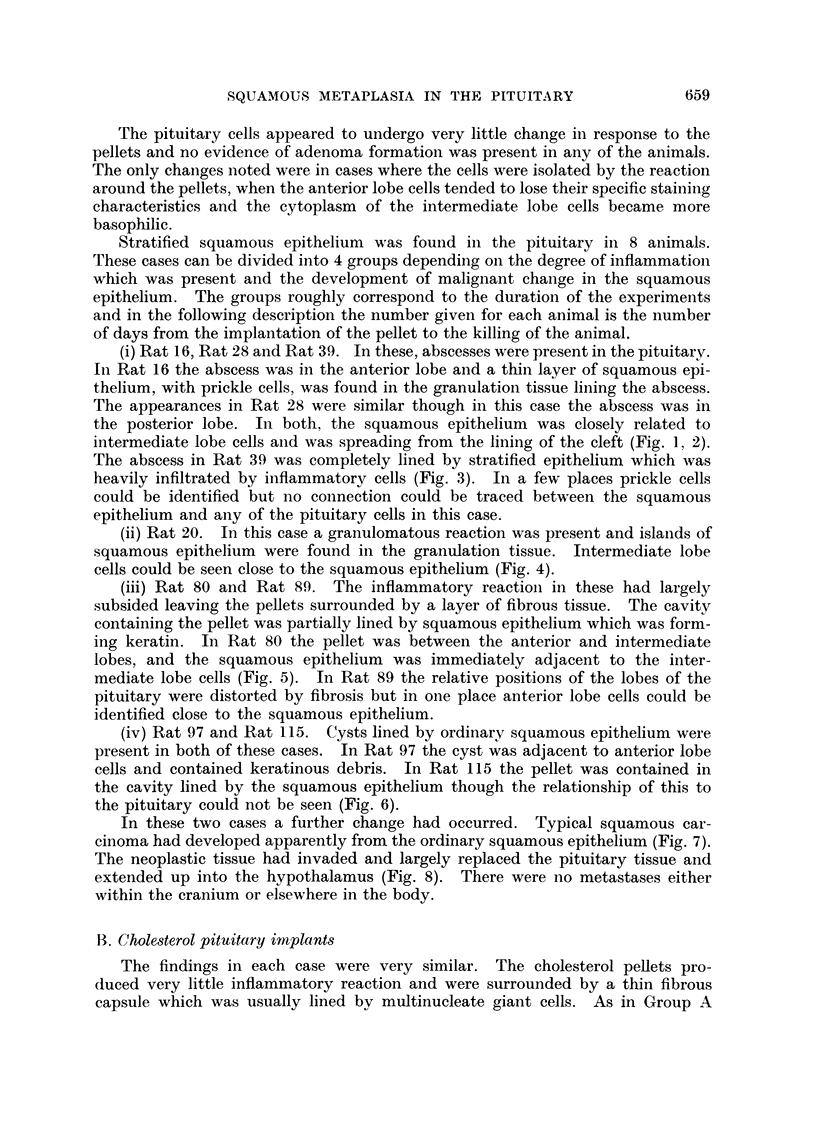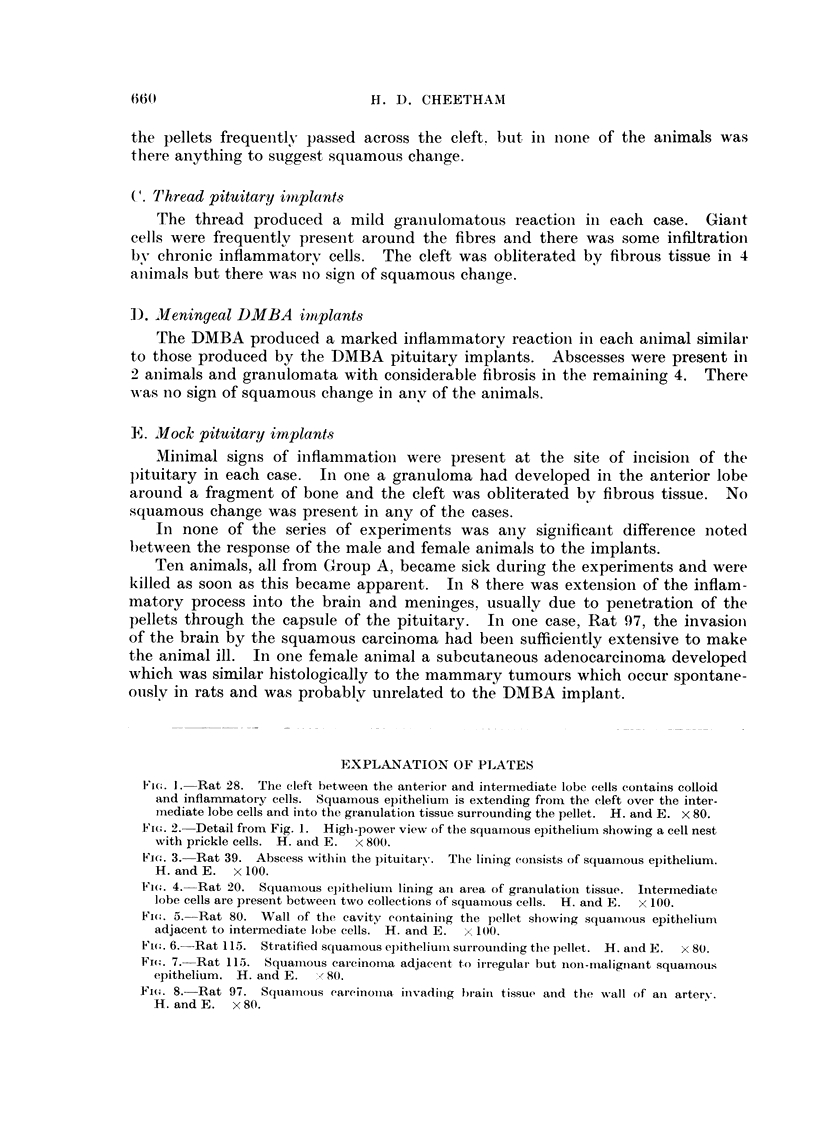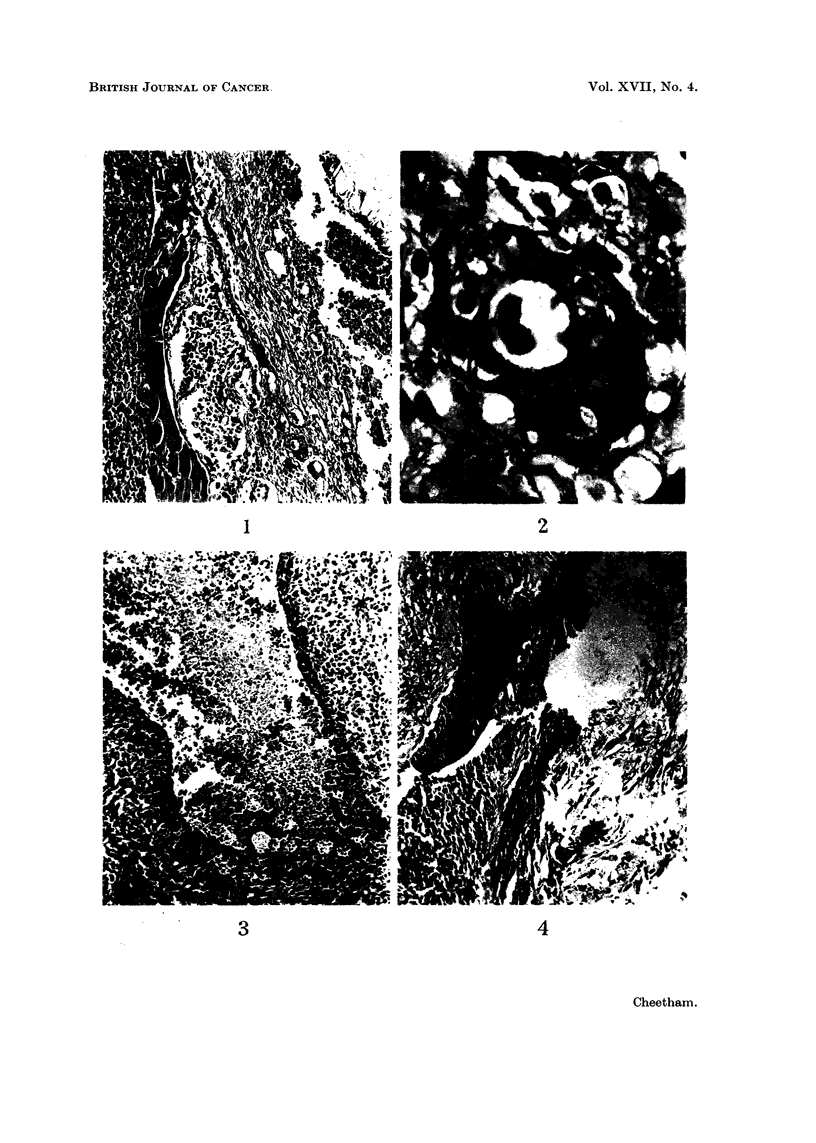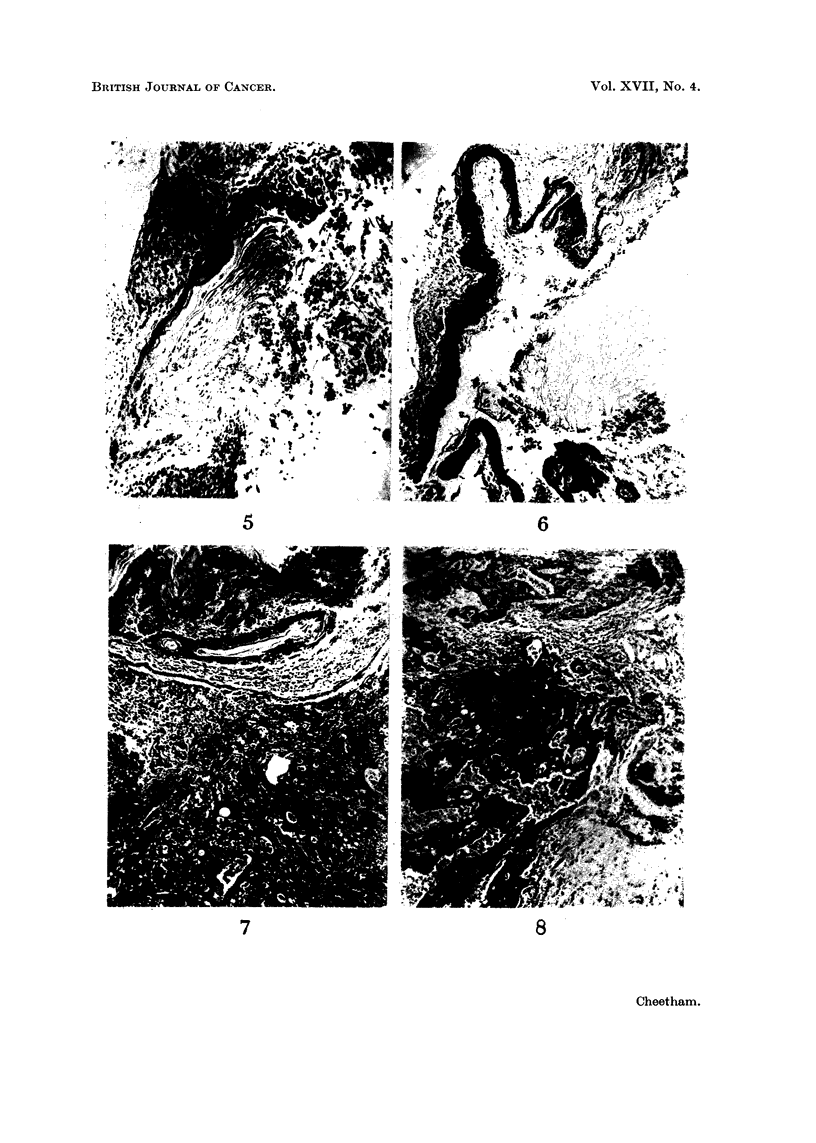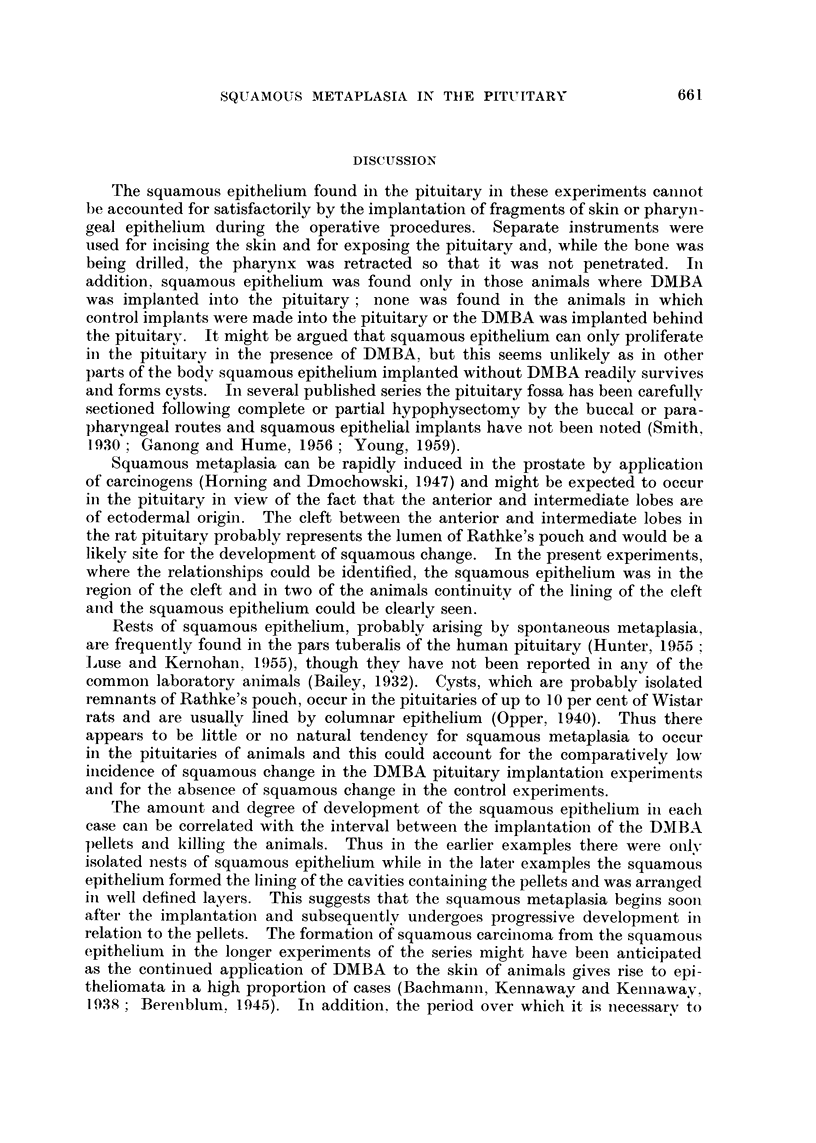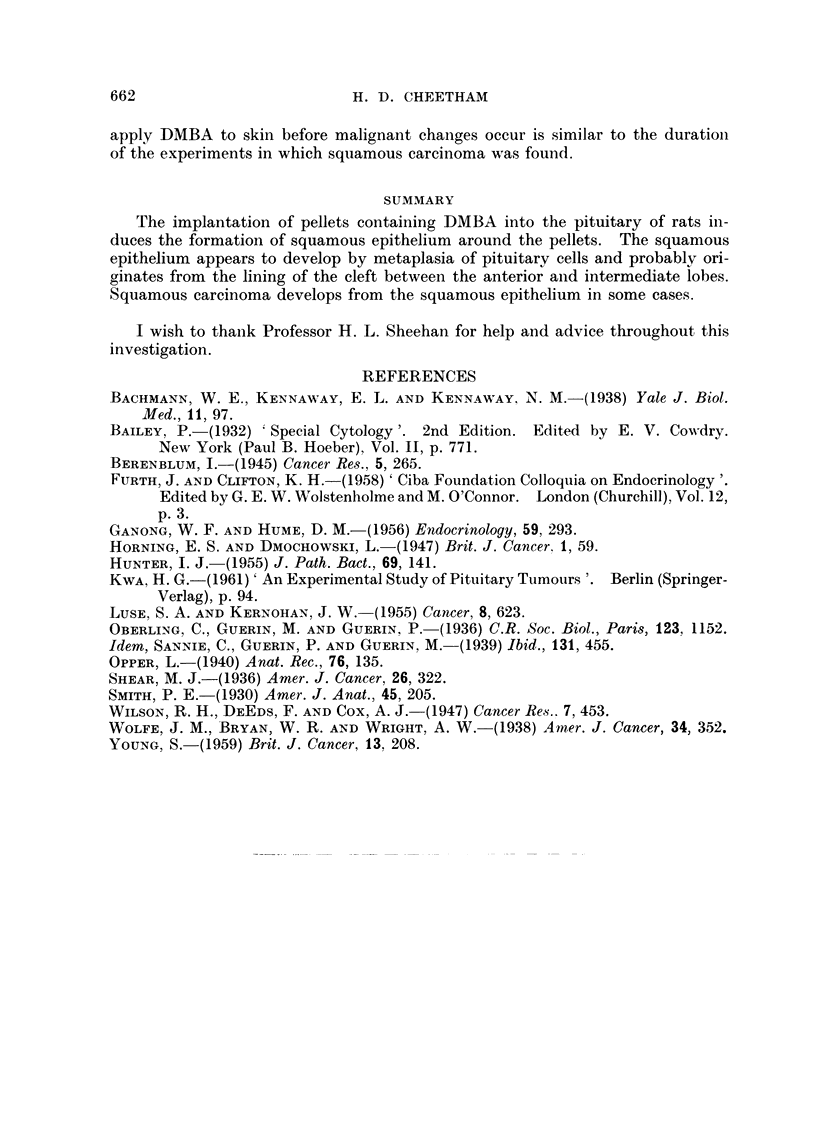# Experimental Squamous Metaplasia and Squamous Epithelioma Formation in the Pituitary of the Rat

**DOI:** 10.1038/bjc.1963.83

**Published:** 1963-12

**Authors:** H. D. Cheetham

## Abstract

**Images:**


### EXPERIMENTAL SQUAMOUS METAPLASIA AND SQUAMOUS EPITHELIOMA FORMATION IN THE PITUITARY OF THE RAT

H. D. CHEETHAM

*From the Endocrine Unit, University of Liverpool**

Received for publication August 1, 1963

THE experimental induction of pituitary tumours has usually involved exposure of animals to ionising radiation or the production of hormonal imbalance in the animals (Furth and Clifton, 1958). The tumours develop from one or other of the main cell types in the pituitary and in some cases there is evidence of functional activity by the tumours, the growth of which may be hormone dependent. Spontaneous pituitary tumours occur particularly frequently in the rat (Wolfe, Bryan and Wright, 1938 ; Oberling, Sannie and Guerin, 1939), and it seems likely that the experimental procedures used to induce the tumours do so by increasing the frequency of the naturally occurring tumours (Kwa, 1961).

The administration of chemical carcinogens to animals, systemically or at sites remote from the pituitary, does not appear to increase the incidence of tumours in the pituitary. In the few reports which have been made, the tumours found were similar to those occurring spontaneously and the incidence was within the range with which these occur naturally (Oberling, Guerin and Guerin, 1936 ; Oberling, Sannie and Guerin, 1939 ; Wilson, DeEds and Cox, 1947). However, in these experiments the concentration of carcinogen acting on the pituitary cells would be relatively low, unless there was special affinity of the carcinogen for the cells. The chance of inducing tumour formation in the pituitary might therefore be increased if the carcinogen was implanted directly into the gland, and the present study was carried out to investigate this possibility.

### METHODS

The rats used were of the Wistar strain and were bred in this laboratory. They were 3–5 months old at the beginning of the experiments and were fed throughout on Oxoid breeding diet 41B and water *ad libitum.*

The animals were anaesthetised with ether and tracheotomised. The base of the skull was exposed by the parapharyngeal approach, as in hypophysectomy, and the fascia connecting the pharynx to the skull was cut, allowing the pharynx to be retracted laterally and improving the exposure. The bone was then drilled, using a 2 mm. dental drill, either at a point centred slightly in front of the spheno-occipital synchrondrosis, to expose the pituitary, or a few mm. behind this point, to expose the meninges. In each case the drill was used until the inner table of bone was reached and this was then broken outwards with a narrow probe. The pituitary implants were made by incising the capsule of the gland with the point of the probe and inserting the material into the gland with fine pointed

* Present address : Department of Bacteriology, University of Liverpool, Liverpool 3.

forceps. The extra-meningeal implants were made by exposing the meninges and forcing the pellets between the meninges and the bone on the side of the hole away from the pituitary. Following the operative procedures the animals were kept in wire mesh cages in groups of not more than four.

Pellets of 9,10-dimethyl-1,2-benzanthracene (DMBA) 5 per cent in cholesterol were prepared by the method of Shear (1936). Those weighing 1 to 5 mg. were selected as being of a size suitable for implantation into the pituitary. Cholesterol pellets were prepared in the same way.

Five series of experiments were carried out.

A. In 20 male and 20 female rats, pellets of DMBA were inserted into the pituitary. The animals were killed in pairs, usually one male and one female, at intervals following the operation. Two pairs were killed during the first week and the remainder at intervals of 8–14 days during the next five months.

B. In 3 male and 3 female rats, pellets of cholesterol were implanted into the pituitary. One male and 1 female rat were killed at monthly intervals.

C. In 3 male and 3 female rats, portions of linen thread were inserted into the pituitary. Linen thread was used as this had been found to produce squamous metaplasia in the salivary gland of the rat (Cruickshank, 1961, personal communication). One male and 1 female rat were killed at monthly intervals.

D. In 3 male and 3 female rats, pellets of DMBA were implanted extradurally behind the pituitary. One male and 1 female rat were killed at monthly intervals.

E. In 3 male and 3 female rats, the pituitary was exposed and the capsule incised but no material was inserted. One male and 1 female rat were killed at monthly intervals.

In each case the animals were killed with ether. The skull was opened and the region of the pituitary carefully inspected. If possible the pituitary was separated from the underlying bone but if this was difficult, or if adhesions were present between the brain and the pituitary, the pituitary, a portion of brain and the bone were removed in one piece. The tissues were fixed in 10 per cent formol saline and, if bone was present, decalcification was carried out in 10 per cent formic acid. Multiple paraffin sections were cut through the tissue and stained with haematoxylin and eosin and in some cases with the Crooke-Russell modification of Mallory's stain.

### RESULTS

### A. *DMBA pituitary implants*

The pellets were highly irritant and signs of inflammation were found in the tissues around the pellet in all the animals. During the first month the changes were usually those of acute inflammation while by the fifth month the pellets were surrounded by dense fibrous tissue and only a few foreign body giant cells and other chronic inflammatory cells were present. The cleft between the anterior and intermediate lobes forms a natural plane of cleavage into which the pellets can pass, and was nearly always involved in the inflammatory reaction. In some cases the epithelium lining the cleft underwent proliferation, and occasionally extended into the underlying tissues. A similar sequence of inflammatory changes takes place when DMBA pellets are implanted into the liver or kidney.

The pituitary cells appeared to undergo very little change in response to the pellets and no evidence of adenoma formation was present in any of the animals. The only changes noted were in cases where the cells were isolated by the reaction around the pellets, when the anterior lobe cells tended to lose their specific staining characteristics and the cytoplasm of the intermediate lobe cells became more basophilic.

Stratified squamous epithelium was found in the pituitary in 8 animals. These cases can be divided into 4 groups depending on the degree of inflammation which was present and the development of malignant change in the squamous epithelium. The groups roughly correspond to the duration of the experiments and in the following description the number given for each animal is the number of days from the implantation of the pellet to the killing of the animal.

(i) Rat 16, Rat 28 and Rat 39. In these, abscesses were present in the pituitary. In Rat 16 the abscess was in the anterior lobe and a thin layer of squamous epithelium, with prickle cells, was found in the granulation tissue lining the abscess. The appearances in Rat 28 were similar though in this case the abscess was in the posterior lobe. In both, the squamous epithelium was closely related to intermediate lobe cells and was spreading from the lining of the cleft (Fig. 1, 2). The abscess in Rat 39 was completely lined by stratified epithelium which was heavily infiltrated by inflammatory cells (Fig. 3). In a few places prickle cells could be identified but no connection could be traced between the squamous epithelium and any of the pituitary cells in this case.

(ii) Rat 20. In this case a granulomatous reaction was present and islands of squamous epithelium were found in the granulation tissue. Intermediate lobe cells could be seen close to the squamous epithelium (Fig. 4).

(iii) Rat 80 and Rat 89. The inflammatory reaction in these had largely subsided leaving the pellets surrounded by a layer of fibrous tissue. The cavity containing the pellet was partially lined by squamous epithelium which was forming keratin. In Rat 80 the pellet was between the anterior and intermediate lobes, and the squamous epithelium was immediately adjacent to the intermediate lobe cells (Fig. 5). In Rat 89 the relative positions of the lobes of the pituitary were distorted by fibrosis but in one place anterior lobe cells could be identified close to the squamous epithelium.

(iv) Rat 97 and Rat 115. Cysts lined by ordinary squamous epithelium were present in both of these cases. In Rat 97 the cyst was adjacent to anterior lobe cells and contained keratinous debris. In Rat 115 the pellet was contained in the cavity lined by the squamous epithelium though the relationship of this to the pituitary could not be seen (Fig. 6).

In these two cases a further change had occurred. Typical squamous carcinoma had developed apparently from the ordinary squamous epithelium (Fig. 7). The neoplastic tissue had invaded and largely replaced the pituitary tissue and extended up into the hypothalamus (Fig. 8). There were no metastases either within the cranium or elsewhere in the body.

B. *Cholesterol pituitary implants*

The findings in each case were very similar. The cholesterol pellets produced very little inflammatory reaction and were surrounded by a thin fibrous capsule which was usually lined by multinucleate giant cells. As in Group A

the pellets frequently passed across the cleft, but in none of the animals was there anything to suggest squamous change.

*C. Thread pituitary implants*

The thread produced a mild granulomatous reaction in each case. Giant cells were frequently present around the fibres and there was some infiltration by chronic inflammatory cells. The cleft was obliterated by fibrous tissue in 4 animals but there was no sign of squamous change.

*D. Meningeal DMBA implants*

The DMBA produced a marked inflammatory reaction in each animal similar to those produced by the DMBA pituitary implants. Abscesses were present in 2 animals and granulomata with considerable fibrosis in the remaining 4. There was no sign of squamous change in any of the animals.

*E. Mock pituitary implants*

Minimal signs of inflammation were present at the site of incision of the pituitary in each case. In one a granuloma had developed in the anterior lobe around a fragment of bone and the cleft was obliterated by fibrous tissue. No squamous change was present in any of the cases.

In none of the series of experiments was any significant difference noted between the response of the male and female animals to the implants.

Ten animals, all from Group A, became sick during the experiments and were killed as soon as this became apparent. In 8 there was extension of the inflammatory process into the brain and meninges, usually due to penetration of the pellets through the capsule of the pituitary. In one case, Rat 97, the invasion of the brain by the squamous carcinoma had been sufficiently extensive to make the animal ill. In one female animal a subcutaneous adenocarcinoma developed which was similar histologically to the mammary tumours which occur spontaneously in rats and was probably unrelated to the DMBA implant.

### EXPLANATION OF PLATES

FIG. 1.—Rat 28. The cleft between the anterior and intermediate lobe cells contains colloid and inflammatory cells. Squamous epithelium is extending from the cleft over the intermediate lobe cells and into the granulation tissue surrounding the pellet. H. and E. ×80.

FIG. 2.—Detail from Fig. 1. High-power view of the squamous epithelium showing a cell nest with prickle cells. H. and E. ×800.

FIG. 3.—Rat 39. Abscess within the pituitary. The lining consists of squamous epithelium. H. and E. ×100.

FIG. 4.—Rat 20. Squamous epithelium lining an area of granulation tissue. Intermediate lobe cells are present between two collections of squamous cells. H. and E. ×100.

FIG. 5.—Rat 80. Wall of the cavity containing the pellet showing squamous epithelium adjacent to intermediate lobe cells. H. and E. ×100.

FIG. 6.—Rat 115. Stratified squamous epithelium surrounding the pellet. H. and E. ×80.

FIG. 7.—Rat 115. Squamous carcinoma adjacent to irregular but non-malignant squamous epithelium. H. and E. ×80.

FIG. 8.—Rat 97. Squamous carcinoma invading brain tissue and the wall of an artery. H. and E. ×80.

1

2

3

4

Cheetham.

5

6

7

8

Cheetham.

### DISCUSSION

The squamous epithelium found in the pituitary in these experiments cannot be accounted for satisfactorily by the implantation of fragments of skin or pharyngeal epithelium during the operative procedures. Separate instruments were used for incising the skin and for exposing the pituitary and, while the bone was being drilled, the pharynx was retracted so that it was not penetrated. In addition, squamous epithelium was found only in those animals where DMBA was implanted into the pituitary ; none was found in the animals in which control implants were made into the pituitary or the DMBA was implanted behind the pituitary. It might be argued that squamous epithelium can only proliferate in the pituitary in the presence of DMBA, but this seems unlikely as in other parts of the body squamous epithelium implanted without DMBA readily survives and forms cysts. In several published series the pituitary fossa has been carefully sectioned following complete or partial hypophysectomy by the buccal or parapharyngeal routes and squamous epithelial implants have not been noted (Smith, 1930 ; Ganong and Hume, 1956 ; Young, 1959).

Squamous metaplasia can be rapidly induced in the prostate by application of carcinogens (Horning and Dmochowski, 1947) and might be expected to occur in the pituitary in view of the fact that the anterior and intermediate lobes are of ectodermal origin. The cleft between the anterior and intermediate lobes in the rat pituitary probably represents the lumen of Rathke's pouch and would be a likely site for the development of squamous change. In the present experiments, where the relationships could be identified, the squamous epithelium was in the region of the cleft and in two of the animals continuity of the lining of the cleft and the squamous epithelium could be clearly seen.

Rests of squamous epithelium, probably arising by spontaneous metaplasia, are frequently found in the pars tuberalis of the human pituitary (Hunter, 1955 ; Luse and Kernohan, 1955), though they have not been reported in any of the common laboratory animals (Bailey, 1932). Cysts, which are probably isolated remnants of Rathke's pouch, occur in the pituitaries of up to 10 per cent of Wistar rats and are usually lined by columnar epithelium (Opper, 1940). Thus there appears to be little or no natural tendency for squamous metaplasia to occur in the pituitaries of animals and this could account for the comparatively low incidence of squamous change in the DMBA pituitary implantation experiments and for the absence of squamous change in the control experiments.

The amount and degree of development of the squamous epithelium in each case can be correlated with the interval between the implantation of the DMBA pellets and killing the animals. Thus in the earlier examples there were only isolated nests of squamous epithelium while in the later examples the squamous epithelium formed the lining of the cavities containing the pellets and was arranged in well defined layers. This suggests that the squamous metaplasia begins soon after the implantation and subsequently undergoes progressive development in relation to the pellets. The formation of squamous carcinoma from the squamous epithelium in the longer experiments of the series might have been anticipated as the continued application of DMBA to the skin of animals gives rise to epitheliomata in a high proportion of cases (Bachmann, Kennaway and Kennaway, 1938 ; Berenblum, 1945). In addition, the period over which it is necessary to apply DMBA to skin before malignant changes occur is similar to the duration of the experiments in which squamous carcinoma was found.

SUMMARY

The implantation of pellets containing DMBA into the pituitary of rats induces the formation of squamous epithelium around the pellets. The squamous epithelium appears to develop by metaplasia of pituitary cells and probably originates from the lining of the cleft between the anterior and intermediate lobes. Squamous carcinoma develops from the squamous epithelium in some cases.

I wish to thank Professor H. L. Sheehan for help and advice throughout this investigation.

REFERENCES

Bachmann, W. E., Kennaway, E. L. and Kennaway, N. M.—(1938) *Yale J. Biol. Med.*, **11**, 97.
Bailey, P.—(1932) 'Special Cytology'. 2nd Edition. Edited by E. V. Cowdry. New York (Paul B. Hoeber), Vol. II, p. 771.
Berenblum, I.—(1945) *Cancer Res.*, **5**, 265.
Furth, J. and Clifton, K. H.—(1958) 'Ciba Foundation Colloquia on Endocrinology'. Edited by G. E. W. Wolstenholme and M. O'Connor. London (Churchill), Vol. 12, p. 3.
Ganong, W. F. and Hume, D. M.—(1956) *Endocrinology*, **59**, 293.
Horning, E. S. and Dmochowski, L.—(1947) *Brit. J. Cancer*, **1**, 59.
Hunter, I. J.—(1955) *J. Path. Bact.*, **69**, 141.
Kwa, H. G.—(1961) 'An Experimental Study of Pituitary Tumours'. Berlin (Springer-Verlag), p. 94.
Luse, S. A. and Kernohan, J. W.—(1955) *Cancer*, **8**, 623.
Oberling, C., Guerin, M. and Guerin, P.—(1936) *C.R. Soc. Biol., Paris*, **123**, 1152.
*Idem*, Sannie, C., Guerin, P. and Guerin, M.—(1939) *Ibid.*, **131**, 455.
Opper, L.—(1940) *Anat. Rec.*, **76**, 135.
Shear, M. J.—(1936) *Amer. J. Cancer*, **26**, 322.
Smith, P. E.—(1930) *Amer. J. Anat.*, **45**, 205.
Wilson, R. H., DeEds, F. and Cox, A. J.—(1947) *Cancer Res.*, **7**, 453.
Wolfe, J. M., Bryan, W. R. and Wright, A. W.—(1938) *Amer. J. Cancer*, **34**, 352.
Young, S.—(1959) *Brit. J. Cancer*, **13**, 208.